# Circulating Resistin Levels and Risk of Colorectal Cancer: A Meta-Analysis

**DOI:** 10.1155/2016/7367485

**Published:** 2016-08-24

**Authors:** Gui Yang, Wei Fan, Baohong Luo, Zhigao Xu, Ping Wang, Shihui Tang, Peipei Xu, Mingxia Yu

**Affiliations:** ^1^Department of Clinical Laboratory, Zhongnan Hospital of Wuhan University, Wuhan 430071, China; ^2^Department of Center for Gene Diagnosis, Zhongnan Hospital of Wuhan University, Wuhan 430071, China; ^3^Department of Pathology, Zhongnan Hospital of Wuhan University, Wuhan 430071, China

## Abstract

*Objectives*. Published data on resistin levels in patients with colorectal cancer (CRC) were conflicting and heterogeneous. We conducted a meta-analysis of observational studies to examine the association of circulating resistin levels with carcinogenesis of the CRC.* Methods*. Potentially eligible studies published up to November 2015 were searched through MEDLINE, EMBASE, Science Citation Index Expanded database, CNKI, and WanFang database. The pooled weighted mean differences (WMDs) with 95% confidence intervals (CIs) calculated by fixed- or random-effect model were used to estimate the effects.* Results*. A total of 11 studies involving 965 patients were admitted in our meta-analysis. The pooled effects indicated that resistin levels were higher in CRC patients compared to healthy controls (WMD: 1.47 ng/mL; 95% CI: 0.78 to 2.16), with significant heterogeneity across the studies (*I* ^2^ = 72%, *p* < 0.0001). Subgroup analyses and sensitivity analyses revealed that study quality, design, sample type, and resistin assays may account for this heterogeneity. No publication bias was observed.* Conclusions*. Our meta-analysis suggests that increased circulating resistin levels are associated with greater risk of colorectal cancer. Given the limited number of available studies and significant heterogeneity, larger well-designed randomized studies are warranted.

## 1. Introduction

Colorectal cancer is the third most common cancer and the third most common cause of high mortality cancer rates in the united states [1]. Although the incidence of CRC is 18% higher in developed regions in comparison with lower-income areas [2], there is a rapid increase in developing countries in the past few decades, which makes it a major public health concern worldwide. CRC is recognized as a complex multipathway disease circled with chronic inflammation, metabolic syndrome, insulin resistance (IR), and obesity. The results from epidemiological evidence indicate that obesity is associated with increased incidence of CRC [3, 4]. Recent studies suggest that adipocyte-derived secretory factors including adiponectin, leptin, resistin, visfatin, and numerous cytokines including TNF-alpha, IL-6, and IL-10 may be the key to link obesity with CRC, although underlying mechanisms remain unexplained [5–8].

Resistin, produced by adipose tissue whereas it is mostly secreted from monocytes and macrophages and involved in autocrine and paracrine cell signaling [9], has raised considerable research interest in recent years [10]. It is a cysteine rich 108-amino-acid peptide with a molecular weight of 12.5 kDa, mapping to the p13.3 band of human chromosome 19 [11, 12] and circulating in low and high molecular weight forms [13]. As a member of resistin-like molecules (RELMs), which is a small family of secreted proteins associated with the activation of inflammatory [14], resistin can regulate inflammatory response, mediate metabolic disturbances and IR, promote cell proliferation, and enhance cancer progression [15], providing novel insights to obesity and colorectal cancer.

Although there are several systematic reviews evaluating the relationship between several adipocytokines and risk of CRC or CRA, including leptin and adiponectin [16–20], to the best of our knowledge, there is no meta-analysis on the associations of circulating resistin and CRC. In the past few years, some studies indicated a positive association between resistin levels and CRC [18, 21–28]; others failed to demonstrate such a relationship [29–32]. Seeing that controversial discrepancy, we summarized the evidence obtained from available data to assess resistin levels in patients with CRC.

## 2. Materials and Methods

Since we were evaluating observational studies, we conducted this meta-analysis in accordance with the Observational Studies in Epidemiology [33].

### 2.1. Search Strategy

A systematic literatures search from online databases including MEDLINE, EMBASE, and ISI Web of Knowledge was performed. The respective scientific studies up to November 20, 2015, were identified without language, year, or publication status restriction. Besides, we searched National Knowledge Infrastructure (CNKI) and WanFang database for Chinese literatures. The following terms were used as strategy: resistin OR adipokine OR adipocytokine OR adipocyte secreted factor (ADSF) OR FIZZ-3 OR obese protein, colorectal OR colon OR rectum, cancer OR tumor OR carcinoma OR neoplasm. Reference lists of identified literature were also checked for additional articles.

### 2.2. Inclusion and Exclusion Criteria

Eligible cohort and case-control studies reporting the association between serum or plasma resistin levels and colorectal cancer were included in this meta-analysis. We excluded studies as follows: (1) reviews or nonoriginal studies; (2) studies on animal or cell or tissue or genetic variation; (3) studies without controls or original data; (4) studies related to mechanism or prognosis or survival.

### 2.3. Data Extraction and Quality Assessment

The following information and data were extracted from each included study: (1) basic information such as first author's name, year of publication, and geographic location; (2) details of each trial, for example, study design and size, type of blood sampling, resistin detection method, and reagent; (3) characteristics of patients and controls including age, gender, BMI estimation, means, and standard deviation (SD) of resistin concentration. Two reviewers independently finished the extraction work and the disagreements were resolved by consensus or the third reviewer. Meanwhile, we systematically assessed the quality of each study with the Newcastle-Ottawa Scale (NOS) [34].

### 2.4. Statistical Analysis

All statistical analyses were conducted with Stata 11.0 (Stata, College Station, Texas, USA) and Review Manager 5.3 software (Cochrane Collaboration). Weighted mean differences (WMDs) and corresponding 95% confidence intervals (CIs) were calculated based on the sample size, mean, and SD extracted from eligible studies. For five studies that only provided the median and range or interquartile range of outcomes, mean and SD were estimated, as described by Hozo et al. [35] and Liu et al. [36]. The Cochran Q statistic and inconsistency index (*I* ^2^) were used for examining the heterogeneity among studies, with the statistical significance level 0.05. Considering the presence of significant heterogeneity, we applied a random effects model for pooling effect, otherwise, a fixed-effects model.

In order to explore the sources of heterogeneity, we performed subgroup analysis by geographical area, study design, study size, quality, blood sample, resistin assay method, mean age, and mean BMI. Besides, several sensitivity analyses were conducted by omitting one study at a time, excluding estimated effect sizes and studies not adjusted for BMI. Publication bias was evaluated by funnel plot, complementary with Egger's regression method and Begg's rank correlation method.

## 3. Results

### 3.1. Search Results and Studies' Characteristics

**Figure 1:**
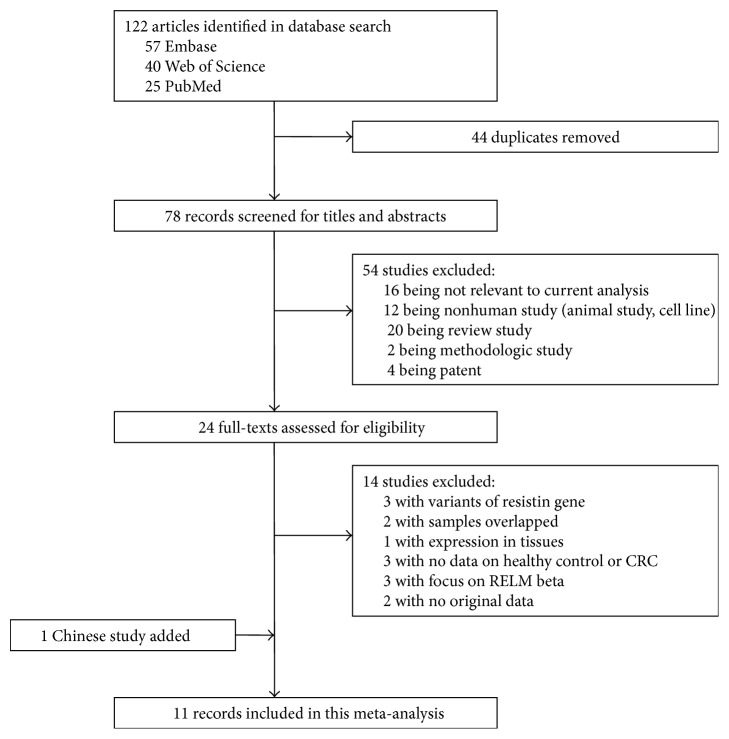
A flow diagram of selection strategy.

A total of 122 potential records were detected by the primary database search and 78 were left after being autodeduplicated. By reading titles and abstracts for relevance, 54 reports were excluded because they were either review articles, mechanism studies, animal studies, or irrelevant to circulating resistin. Of the 24 records selected for detailed full-text evaluation, only 10 studies fulfilled our inclusion criterion. Three studies did not measure circulating resistin levels in healthy controls. Two trials were excluded because of one provided box-plot without data to describe distribution of serum resistin concentration and the other reported only odd ratio (OR) and 95% CI of the risk of colorectal cancer; we could not get primary data although we tried to contact the authors. Besides, a Chinese study that met the inclusion criterion was added. A flow diagram of selection strategy was presented in Figure 1.

Consequently, 11 studies including 965 patients with colorectal cancer and 1325 healthy controls were identified in this meta-analysis. Among the 11 studies, one was case-cohort study conducted in USA [31], while the other 10 studies were of case-control design conducted either in Asia [18, 21, 23, 25, 26, 28] or in Europe [24, 27, 29, 32]. Ten of the studies were matched for age and/or gender [18, 21, 23, 24, 26–29, 31, 32] and 6 of them were adjusted for BMI additionally [21, 23, 24, 26, 27, 31]. The characteristics of each study were summarized in Table 1. The quality assessments of 11 case-control studies were presented in Table 2. The only case-cohort study [31] was finally awarded 7 stars based on Newcastle-Ottawa Scale (NOS) and specific assessment items were not listed.

### 3.2. Data Analysis

**Figure 2:**
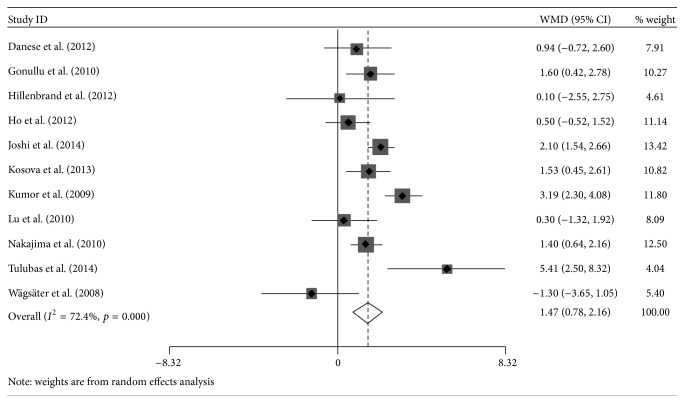
Forest plot of weighted mean differences (WMD) with 95% confidence intervals (CIs) of circulating resistin levels between colorectal cancer patients and healthy controls.

Three studies showed that patients with colorectal cancer had no significant difference in circulating resistin concentration compared to healthy controls [29, 31, 32], while significant increase was presented in other nine studies [18, 21–28]. The summary WMD of resistin levels between CRC patients and control group was 1.47 ng/mL (95% CI: 0.78 to 2.16), and *z*-score for overall effect was 4.19 (*p* < 0.0001), which revealed a possibility that higher circulating resistin level was associated with higher risk of colorectal tumor. Significant heterogeneity was observed among studies and needed further analysis (*I* ^2^ = 72%, *p* < 0.0001; Figure 2).

### 3.3. Subgroups/Sensitivity Analysis

We subsequently conducted subgroups and sensitivity analysis to trace the potential sources of heterogeneity. In subgroup analysis, the summary WMD of circulating resistin levels between CRC patients and healthy controls did not differ substantially by mean age, sample size, and mean BMI. Thus, heterogeneity could not be explained by those variables. However, positive association was observed between resistin levels and risk of colorectal cancer in serum samples, studies with ELISA method, low quality studies, studies of Asian origin, and case-control design, indicating the possible origin of heterogeneity. Pooled WMD of resistin levels in serum samples (WMD, 1.97; 95% CI, 1.18–2.75), studies with ELISA method (WMD, 1.68; 95% CI, 0.95–2.41), low quality studies (WMD, 1.69; 95% CI, 0.98–2.41), studies of Asian origin (WMD, 1.70; 95% CI, 1.03–2.37), and case-control design (WMD, 1.84; 95% CI, 1.51–2.18) were stronger than in plasma samples (WMD, 0.59; 95% CI, −0.39–1.57), studies with multiplex immunoassay (WMD, 0.45; 95% CI, −0.50–1.40) and high quality studies (WMD, 1.22; 95% CI, −0.33–2.78), studies of Western origin (WMD, 0.93; 95% CI, −1.20–3.05), and case-cohort design (WMD, 0.50; 95% CI, −0.52–1.52). Results of subgroups analysis are presented in Table 3.

**Figure 3:**
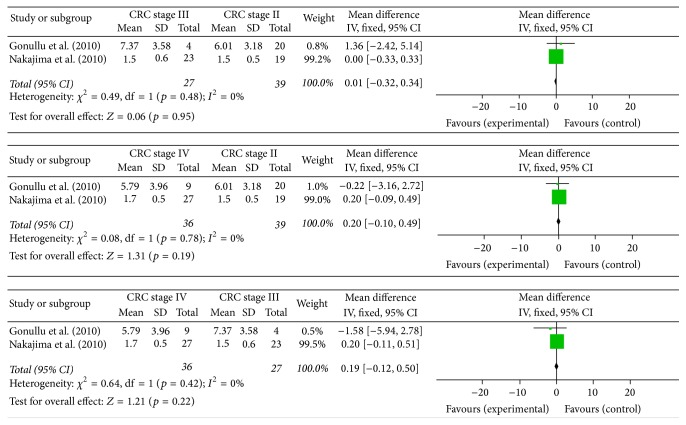
Forest plot of mean differences with its 95% CIs of circulating resistin levels in CRC stage II–IV patients.

Three of 11 studies mentioned the relationship between circulating resistin levels and TNM stages of colorectal cancer. The results of those studies were inconsistent (Table 4). Pooled mean differences suggested that circulating resistin levels might not be significantly different in CRC stage II–IV patients (Figure 3).

**Figure 4:**
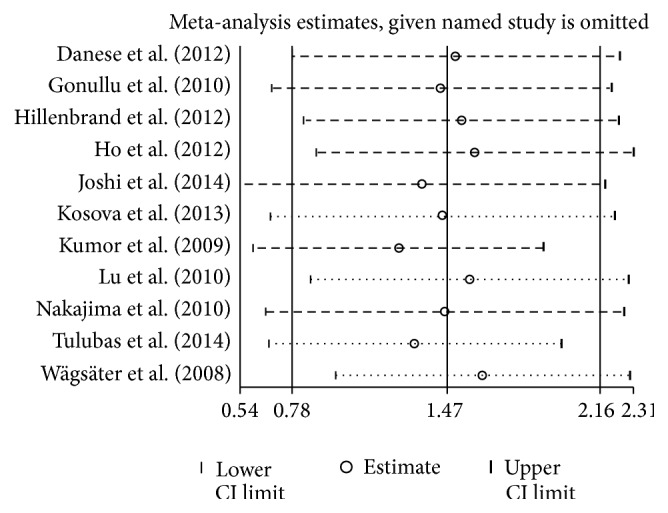
Influence analysis in studies of circulating resistin and colorectal cancer.

In sensitivity analysis, there was no significant difference after deleting a single study at a time and calculating pooled WMD of the remainders (Figure 4). Neither separate analysis of studies matching for BMI yielded significant associations of resistin levels and colorectal cancer risk. While removing studies for which the mean and SD were estimated [23, 27, 29, 31, 32], a stronger association between resistin and CRC was noted (WMD, 2.10; 95% CI, 1.25–2.95).

### 3.4. Publication Bias

**Figure 5:**
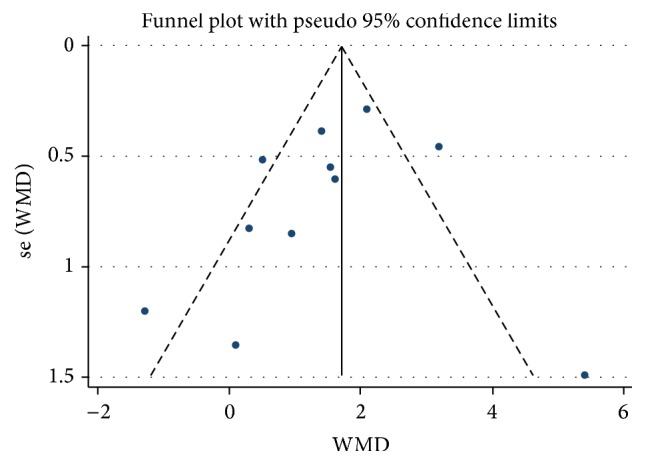
Funnel plot of publication bias.

**Figure 6:**
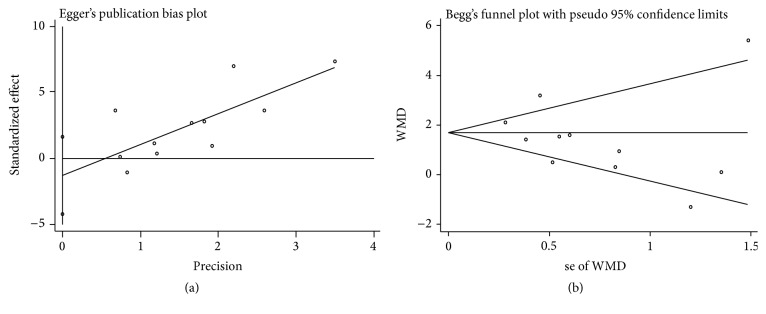
Egger's publication bias plot (a) and Begg's funnel plot (b).

We did not find distinct funnel plot asymmetry, raising a low probability of publication bias (Figure 5). Egger's regression test (*p* = 0.34) and Begg's adjusted rank correlation test (*p* = 0.78) verified that possibility (Figure 6).

## 4. Discussion

The results of this meta-analysis suggested that resistin levels were notably higher in patients with CRC than those in healthy controls, indicating that resistin levels may be positively correlated with risk of colorectal cancer, although there was significant heterogeneity among the studies. The effects of estimates were consistent for studies regardless of their sample size, mean age, mean BMI, and whether being adjusted for BMI or not. No publication bias was observed in our meta-analysis. Keeping in mind the role of resistin in linking obesity to colorectal cancer risk, as well as the inconsistent published results regarding the effect of resistin in this setting, this meta-analysis is of special importance.

Although the role of resistin in colorectal cancer is far from being elucidated, several mechanisms may be involved in explaining these outcomes. In vitro studies demonstrate that resistin has proinflammatory effects mediated by TLR4 receptor stimulation and NF-*κ*Β pathway [37, 38]. Other studies show that resistin regulates production of the matrix metalloproteinases (MMPs) and modulates the secretion of vascular endothelial growth factor (VEGF), which is considered of importance for promoting tumor invasion [39, 40]. An animal study, where the mice were dealt with to lack adipocyte-derived mouse resistin but be able to produce human resistin resulting in rapidly accelerated white adipose tissue (WAT) inflammation after the high-fat diet, indicated that resistin contributes to WAT inflammation and insulin resistance [41]. Furthermore, RETN (rs1862513) or -420C>G resistin gene polymorphism is associated with decreased CRC susceptibility [29, 42].

Recently, studies demonstrate that obesity is strongly related to CRC, which is involved in several mechanisms, such as obesity-related insulin resistance, oxidative stress [43], adipocytokine production [44], and insulin-like growth factor-1 (IGF-1) [45]. They are all responsible for cancer promoting effects, increasing tumor cell migration, and finally leading to tumor metastasis. The overexpression of resistin results in the accumulation of intracellular lipid and then leads to obesity-mediated insulin resistance and inflammation [46, 47]. Therefore, it is widely accepted that high resistin levels are related to inflammatory conditions and malignancies [48, 49].

However, we cannot ignore the significant heterogeneity across studies in the interpretation of the findings. To investigate potential sources, we performed subgroup and sensitivity analyses. Our study suggested that BMI may not be important in explaining the heterogeneity, since effect estimates showed no difference in different mean BMI subgroup and studies whether adjusting for BMI or not. However, the stronger and more detailed estimating factor for obesity including waist circumference and waist-to-hip ratio was not available in most involved studies, so effect of visceral fat accumulation could not be checked. Furthermore, cachexia patients with advanced CRC have lower BMI, which may be a confounding factor. Notably, the low quality and case-control studies pooled stronger relationship between resistin levels and CRC. The only case-cohort study with high quality demonstrated that plasma level of resistin was not associated with CRC. The discrepancy highlights the need for well-designed prospective studies on resistin and colorectal cancer. Besides, sample type and detection method may also contribute to heterogeneity. It may be explained by the reason that circulating resistin levels are affected by different storage condition and sensibility of assays referring to the very low resistin concentration in human blood.

There are other limits which should be acknowledged in our analysis. First, because this meta-analysis is based on observational studies that have inherent bias, the causal relationship of higher resistin levels and higher risk of CRC may not exist. Second, we eventually included 11 studies and 965 CRC patients in our analysis encompassing a case-cohort study which consisted of almost half research patients; the numbers of studies classified into some subgroups were too small to perform a pooled analysis. Possibly because resistin is newly discovered, researches with large sample size are lacked. Third, the estimates of mean and SD from median and range are a cause of bias as described in sensitivity analysis results. At last, we did not rule out residual confounders that may alter resistin levels, including lifestyle parameters such as smoking, alcohol, exercise, nutritional habits, and clinicopathological features such as tumor stage, localization, and type.

In conclusion, our study suggests that circulating resistin levels in patients with CRC are significantly higher than those in healthy controls, increasing the current understanding of the association between resistin levels and risk of colorectal cancer. Given the limitations mentioned above, larger well-designed randomized studies and even experimental researches are needed to confirm the effect of resistin levels in the development of colorectal cancer.

## Figures and Tables

**Table 1:** Characteristics of the studies included in meta-analysis.

References	Country	Blood sample	Study design	Case/control, number	Assay method and resource	Assay resource	Total resistin means (ng/mL) and SD
Kumor et al. 2009 [24]	Poland	Serum	Case-control	36/25	ELISA	R&D Systems	6.79 ± 2.41/3.6 ± 1.08
Gonullu et al. 2010 [26]	Turkey	Serum	Case-control	36/37	ELISA	BioSource	6.1 ± 3.3/4.5 ± 1.5
Nakajima et al. 2010 [23]	Japan	Plasma	Case-control	115/115	ELISA	BioVender Laboratory Medicine	4.5 (3.1–6.4)/3.1 (2.2–4.7)^a^
Danese et al. 2012 [27]	Italy	Serum	Case-control	40/40	ELISA	Mediagnost	5.88 (1.84–22.35)/4.94 (1.82–8.16)^b^
Hillenbrand et al. 2012 [32]	Germany	Plasma	Case-control	67/60	Multiplex immunoassay	Millipore	10.9 (4.2–43.5)/10.8 (5.2–24.9)^b^
Ho et al. 2012 [31]	USA	Plasma	Case-cohort	456/834	Multiplex immunoassay	Millipore	12.8 (9.9–16.4)/12.3 (9.8–15.6)^a^
Joshi et al. 2014 [18]	South Korea	Serum	Case-control	100/100	ELISA kit	Adipogen	4.9 ± 2.3/2.8 ± 1.7
Lu et al. 2010 [28]	China	Serum	Case-control	30/30	ELISA	ADL	7.72 ± 2.6/7.42 ± 3.72
Tulubas et al. 2014 [21]	Turkey	Serum	Case-control	30/30	ELISA	AssayMax ELISA kit	18.77 ± 5.09/13.36 ± 6.36
Wägsäter et al. 2008 [29]	Sweden	Plasma	Case-control	35/34	ELISA	R&D Systems	3.95 (1.50–2.40)/5.25 (2.00–3.00)^b^
Kosova et al. 2013 [25]	Turkey	Serum	Case-control	20/20	ELISA	Millipore Corporation	4.92 ± 2.2/3.39 ± 1.1

^a^Median (interquartile range).

^b^Media (range).

**Table 2:** Assessment of study quality.

Quality indicators from Newcastle-Ottawa Scale	Kumor et al. [24]	Gonullu et al. [26]	Nakajima et al. [23]	Danese et al. [27]	Joshi et al. [18]	Lu et al. [28]	Tulubas et al. [21]	Wägsäter et al. [29]	Kosova et al. [25]	Hillenbrand et al. [32]
Independent validation of case definition	Yes	Yes	Yes	Yes	Yes	No	Yes	Yes	No	No
Consecutive or obviously representative series of cases	No	No	Yes	No	No	No	Yes	Yes	Yes	No
Community controls	No	No	No	No	No	No	No	Yes	No	No
Controls have negative colonoscopy	Yes	Yes	Yes	Yes	No	No	Yes	No	No	No
Study controls for age/gender	Yes	Yes	Yes	Yes	Yes	Yes	Yes	Yes	No	Yes
Study controls for BMI	Yes	Yes	Yes	Yes	No	No	Yes	No	No	No
Secure record of resistin levels with blinded interview	No	No	No	No	No	No	No	No	No	No
The same method of resistin measurement for cases and controls	Yes	Yes	Yes	Yes	Yes	Yes	Yes	Yes	Yes	Yes
The same nonresponse rate for cases and controls	No	No	No	No	No	No	No	No	No	No
Score	5	5	6	5	3	2	6	6	2	2

**Table 3:** Subgroup analysis of circulating resistin levels and colorectal cancer.

Stratification group	Data points (*N*)	Cases		Random effects	Heterogeneity	
		CRCs	Controls	WMD (95% CI)	*I* ^2^ (%)	*p* value
All studies	11	965	1325	1.47 [0.78, 2.16]	72	<0.0001
Study location						
Western	4	178	159	0.93 [−1.20, 3.05]	83	0.0005
Asian	6	331	332	1.70 [1.03, 2.37]	57	0.04
USA	1	456	834	0.50 [−0.52, 1.52]	—	—
Study design						
Case-control	10	509	491	1.84 [1.51, 2.18]	70	0.0004
Case-cohort	1	456	834	0.50 [−0.52, 1.52]	—	—
Sample size						
<100 CRC cases	8	294	276	1.48 [0.38, 2.57]	75	0.0002
≥100 CRC cases	3	671	1049	1.42 [0.55, 2.28]	74	0.02
Blood sample						
Plasma	4	673	1043	0.59 [−0.39, 1.57]	50	0.11
Serum	7	292	282	1.97 [1.18, 2.75]	68	0.004
Assay method						
ELISA	9	442	431	1.68 [0.95, 2.41]	72	0.0004
Multiplex immunoassay	2	523	894	0.45 [−0.50, 1.40]	0	0.78
Study quality						
Low (NOS score ≤ 5)	7	329	312	1.69 [0.98, 2.41]	62	0.01
High (NOS score > 5)	4	636	1013	1.22 [−0.33, 2.78]	79	0.002
Mean age, years						
<60	2	66	67	1.15 [0.19, 2.11]	38	0.2
≥60	9	879	1238	1.56 [0.65, 2.47]	79	<0.0001
Not mentioned	1	20	20	1.53 [0.45, 2.61]	—	—
Mean BMI, kg/m^2^						
<25	1	115	115	1.40 [0.64, 2.16]	73	0.01
≥25	6	665	1026	1.83 [0.54, 3.12]	79	0.0002
Not mentioned	4	185	184	1.03 [−0.14, 2.21]	73	0.01

**Table 4:** Circulating resistin levels in TNM patients with colorectal cancer.

Studies	Sample size	Stage 0	Stage I	Stage II	Stage III	Stage IV	*p* value	Detection methods
Nakajima et al. 2010 [23]	115	1.3 ± 0.5	1.6 ± 0.5	1.5 ± 0.5	1.5 ± 0.6	1.7 ± 0.5	<0.01	ELISA
Gonullu et al. 2010 [26]	33	—	—	6.01 ± 3.18	7.37 ± 3.58	5.79 ± 3.96	—	ELISA
Kumor et al. 2009 [24]	36	—	5.86 ± 3.1		6.79 ± 2.41		>0.05	ELISA
